# Advancing brain-machine interfaces: moving beyond linear state space models

**DOI:** 10.3389/fnsys.2015.00108

**Published:** 2015-07-28

**Authors:** Adam G. Rouse, Marc H. Schieber

**Affiliations:** ^1^Department of Neurology, University of RochesterRochester, NY, USA; ^2^Department of Neurobiology and Anatomy, University of RochesterRochester, NY, USA; ^3^Department of Biomedical Engineering, University of RochesterRochester, NY, USA

**Keywords:** brain-computer interface, hand, kinematic synergy, motor cortex, movement phase, muscle synergy, neuroprosthetics, null space

## Abstract

Advances in recent years have dramatically improved output control by Brain-Machine Interfaces (BMIs). Such devices nevertheless remain robotic and limited in their movements compared to normal human motor performance. Most current BMIs rely on transforming recorded neural activity to a linear state space composed of a set number of fixed degrees of freedom. Here we consider a variety of ways in which BMI design might be advanced further by applying non-linear dynamics observed in normal motor behavior. We consider (i) the dynamic range and precision of natural movements, (ii) differences between cortical activity and actual body movement, (iii) kinematic and muscular synergies, and (iv) the implications of large neuronal populations. We advance the hypothesis that a given population of recorded neurons may transmit more useful information than can be captured by a single, linear model across all movement phases and contexts. We argue that incorporating these various non-linear characteristics will be an important next step in advancing BMIs to more closely match natural motor performance.

## Introduction

Research and development of brain-machine interfaces (BMIs) to restore lost motor function has expanded dramatically in recent years. Whereas, not long ago the state of the art in both non-human primates (Serruya et al., 2002; Taylor et al., 2002) and humans (Hochberg et al., 2006) consisted of controlling a cursor on a computer screen, recent advances in restoring upper limb function have incorporated robotic arms with the ability to grip and manipulate objects using either virtual (Carmena et al., 2003; Chadwick et al., 2011) or robotic (Velliste et al., 2008; Hochberg et al., 2012; Collinger et al., 2013; Wodlinger et al., 2015) hands and digits. In addition to restoring upper limb function, innovative exoskeletons are being used to restore trunk and leg function (Fitzsimmons et al., 2009; Lebedev and Nicolelis, 2011). Functional electrical stimulation also has made possible restoration of movement of the subject's own limbs (Moritz et al., 2008; Pancrazio and Peckham, 2009; Ethier et al., 2012). Many of these advances have transitioned from the research laboratory with non-human primates to the clinical world with human subjects.

Different but inter-related improvements have contributed to these advances. Better recording systems that allow chronically implanted electrodes to record an increasing number of channels of neural activity simultaneously have had a major impact (Stieglitz et al., 2009; Homer et al., 2013). Better understanding of how to extract various neural signals, paradoxically including less focus on precise spike sorting and more use of simple detection methods like threshold crossings (Fraser et al., 2009; Chestek et al., 2011; Hochberg et al., 2012; Wodlinger et al., 2015), the application of statistical models to better estimate firing rates (Cunningham et al., 2010), and the implementation of Kalman filtering (Wu et al., 2003; Li et al., 2009), all have improved BMI performance as well. Better appreciation of the neural adaptation that occurs under BMI conditions has led to dynamic updating of decoding algorithms that enable BMI learning to be both faster and more robust to external changes (Gilja et al., 2012; Orsborn et al., 2012; Zhang and Chase, 2013; Shanechi et al., 2014). A focus on overcoming the technical challenges of chronically monitoring larger and larger amounts of neural activity and on controlling increasingly complex devices has advanced the field substantially and quickly.

Over the same period during which these advances have been made, relatively little has changed in how our understanding of natural motor physiology is applied to BMI control. Current BMI designs almost always assume that neural encoding is a linear, time-invariant system with independent degrees of freedom (DOFs), and therefore implement control algorithms that map neural inputs to a constant set of output variables with a fixed gain. Yet neural control of mammalian motor systems and the behaviors they produce cannot be explained fully with such an idealized model.

Here we examine selected aspects of motor behavior and physiology to explore ways in which current scientific understanding might be exploited to advance the design of BMIs toward achieving performance closer to that of natural human movement. We discuss four different topics. First, we consider differences in motor performance for very fast or very slow movements and examine how BMI decoding might better emulate similar principles. Second, we address evidence that cortical activity—even in areas with significant spinal projections—differs considerably from a veridical representation of actual movement of the body and we consider the implications of these differences for continuous BMI operation. Third, we explore the evidence that natural movement is not statistically independent across different joints or muscles but rather is coordinated and we ask how this might be incorporated more extensively in BMI design. And fourth, we explore the implications of having a large neuronal population to generate movement, examining whether such a system can be modeled effectively with a single, linear state space, and asking whether adhering to such a model has become a limiting oversimplification in current BMI designs. Although the four topics we explore may appear diverse and unrelated, they share the common theme that incorporating a deeper understanding of the non-linear dynamics of normal motor behavior and physiology—during different phases of movement and in different contexts—can advance BMI design. Whereas, scientists generally seek to identify the simplest explanation for the largest set of observations and engineers seek to provide the simplest design to achieve a specific function (Müller et al., 2003), here we argue that the next wave of advances in BMI technology will require incorporating additional levels of complexity.

## Dealing with a wide dynamic range of movement

Humans are capable of performing skilled movements on a wide range of spatial and temporal scales, from the athletic prowess of throwing or kicking a ball at speeds approaching 100 miles per hour (44.7 m/s) to the fine motor skills required for watchmaking and surgery. But throwing a ball is not necessarily controlled in the same fashion as knotting a suture. We therefore consider how control might vary depending on the extent to which a subject intends to make a gross movement quickly vs. a fine movement accurately.

### Dynamic range of movements

In natural movements, Fitts's law (Fitts, 1954) describes a tradeoff between speed and accuracy: The faster the movement, the less accurate it will be; greater accuracy is achieved with slower movement. This principle has been documented in numerous natural movement tasks and under many different conditions (Card et al., 1978; Jagacinski and Monk, 1985; Epps, 1986; MacKenzie, 1992), in neural correlates (Ifft et al., 2011), and in BMI tasks with direct neural control (Felton et al., 2009).

Current BMIs, however, do little to emulate the robust range of behavior observed to follow Fitts's law for either very fast or very accurate movements, relying instead on the designer to pick a fixed gain between input neural signals and output magnitude. This practice effectively limits good BMI performance to a narrow range of the speed/accuracy trade-off. Enabling the user's neural activity to select the movement speed and the associated accuracy dynamically depending on the current phase or context of the task could improve BMI control, providing a range of performance closer to that of natural movements.

### Improving the precision of BMIs

Fine, skilled movements presumably require more precise neural control signals than gross movements, whether the control signal is encoding muscle activation, force, joint position, or velocity. A study by Slifkin and Newell (1999) found, for example, that whereas the average maximum voluntary contraction (MVC) of the index finger against a load cell was 31.07 N with a standard deviation of ~2.1 N, when producing forces of 5% MVC (~1.55 N) the standard deviation was only ~0.09 N. Variability in normal human force production thus scales with the magnitude of force being produced. Such signal-dependent noise appears in many natural motor behaviors (Harris and Wolpert, 1998). Smaller movements are made with smaller errors.

A system that linearly transforms an input to output with constant noise cannot be optimized for fine, accurate movement and for gross, fast movement simultaneously. Indeed, cortical microcircuits in the motor system recently have been proposed to adjust neuron tuning according to the level of precision required. By functionally varying the overall strength of excitatory/inhibitory drive and changing the tuning widths of individual M1 neurons, the accuracy and precision of movement encoding by a population of neurons might be adjusted dynamically (Mahan and Georgopoulos, 2013; Georgopoulos, 2014). Although natural adjustment of neuronal tuning may be present in the input neurons, current BMIs that rely on the linear sum of neural activity nevertheless show poor adjustment of precision compared to that found in natural movements. Such suboptimal adjustment of precision might result from the small number of input neurons compared to the natural motor system and/or the lack of adjustment for precision in the tuning and decoding models used currently.

**Figure 1:**
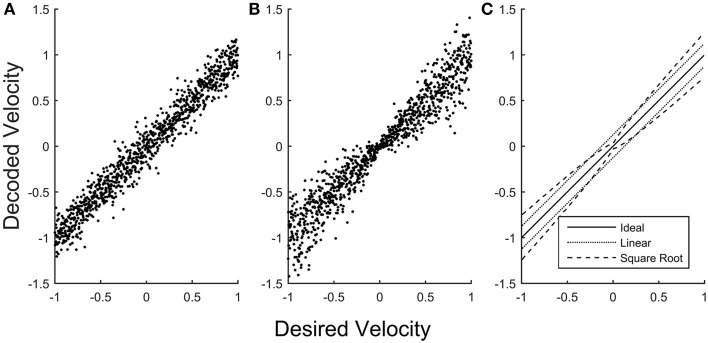
**Error in simulated velocity decoding**. A simulated population of neurons was scaled to encode velocities (with a maximum system velocity equal to 1) using two different methods. **(A)** The simulated population encodes linear velocity, producing uniform error in the decoded velocity that is independent of the desired velocity. **(B)** The simulated population (with the same dynamic range and noise properties of the neurons) is now scaled to encode the square root of velocity, producing error in the decoded velocity that becomes smaller for velocities closer to zero. **(C)** The standard deviation of decoded velocity is plotted as a function of desired velocity for the linear model (**A**, dotted) and for the square root model (**B**, dashed), emphasizing that when decoding linear velocity the error is constant across desired velocities, whereas for the square root model the error is smaller close to zero velocity and larger for higher velocities.

BMIs therefore might be improved by creating decoding schemes that more explicitly allow for encoding of the speed-accuracy tradeoff from a recorded subpopulation. If we construct a BMI model that uses instantaneous neuronal firing rates to encode velocity in a single dimension with a simple linear encoder, the magnitude of the error is uniform for all encoded velocities (Figure 1A). If instead the neurons in the model are assigned to encode a rescaling of velocity, the square root of velocity for example, the errors near zero velocity are smaller than those for high velocity (Figure 1B), more closely emulating the signal-dependent noise of natural behavior (Figure 1C). Applying such approaches to create noise profiles that more closely match natural behavior is likely to create BMI controlled movements that appear more natural by allowing greater precision for fine movements.

## The relationship of cortical activity to physical movement

The majority of current BMIs that control motor output use neural activity recorded from motor and premotor areas of the cerebral cortex as input for two reasons. First, many aspects of natural movements are represented in the activity of neurons in these cortical areas. Second, the cerebral cortex can be accessed for neural recording relatively easily compared to deeper parts of the brain and spinal cord. But using only cortical activity has other advantages and disadvantages for BMI control: (i) certain features of cortical activity are not output to physical movement of the body, (ii) the tuning of cortical neurons changes when controlling a BMI, and (iii) certain aspects of physical movement may not be controlled directly from the cortex.

### Motor imagery, mirror neurons, and BMI control

During motor imagery, when humans imagine themselves performing movements without actually making any movement, activation appears in many of the same cortical motor areas that are activated during physical movement performance, including the primary motor cortex (Ersland et al., 1996; Grafton et al., 1996; Porro et al., 1996; Pfurtscheller and Neuper, 1997; Anderson et al., 2011; Ajiboye et al., 2012). Likewise during action observation, when a monkey observes another individual performing a particular movement, a large subpopulation of neurons discharges in a fashion similar to their discharge when the monkey executes the same action itself. These “mirror neurons,” as well as other neurons that are activated when the monkey observes a cursor moved by the investigator, have been observed not only in premotor cortex but in primary motor cortex as well (Cisek and Kalaska, 2004; Tkach et al., 2007; Dushanova and Donoghue, 2010; Casile, 2013; Vigneswaran et al., 2013). Many neurons in motor cortex thus discharge during motor imagery and/or action observation, when subjects are not making any physical movement.

BMI experiments with normal subjects are perhaps the most clear-cut demonstration that neurons active during natural movements can also be activated voluntarily in the absence of physical movement. In such BMI experiments, normal monkeys typically first perform a task involving physical movement of their native limb while neural activity is recorded from the cortex. This activity then is used to calibrate a decoding algorithm that relates the recorded neuronal activity to the observed limb movements. But after switching to “brain-control”—now controlling a cursor or prosthesis directly through the decoding algorithm—the monkey often stops making any physical movement or producing any EMG activity, even though allowed to move freely (Taylor et al., 2002; Carmena et al., 2003). Meanwhile, the recorded neurons continue to modulate, voluntarily controlling the external BMI device. Like mirror neurons, these neurons are activated during physical movement of the native limb, but are not obligatorily coupled to physical movement. Though in most BMI experiments neurons have not been tested for such, many of them might have activity during action observation.

Indeed, we know that neurons activated during action observation can be used for voluntary BMI control, because in both normal monkeys and paralyzed humans neuronal activity recorded during action observation can be used effectively to develop the initial decoding algorithm (Velliste et al., 2008; Chadwick et al., 2011; Collinger et al., 2013). Although using neurons activated by action observation currently provides an advantage for calibrating BMIs in paralyzed or amputated subjects, using mirror neurons might have disadvantages as well. In one recent study, for example, having a particular object present during the initial calibration was needed to enable the subject subsequently to close the prosthetic hand optimally around that object (Wodlinger et al., 2015), possibly indicating that the recorded population included a substantial number of mirror neurons encoding that particular grasp shape. More detailed scientific understanding of the differences in neuronal activity during motor imagery vs. action observation vs. action execution may enable BMI decoding based on these differences to improve performance.

### Changes in cortical activity during BMI control

The change from “hand-control” to “brain-control” changes cortical activity. Many neurons, for example, show changes in their directional tuning (Taylor et al., 2002; Ganguly and Carmena, 2009). Such changes may result from alterations in proprioceptive feedback because a normal subject's native limb that moved during hand-control either moves differently or does not move at all during brain-control. Differences may exist as well in the descending regulation of sensory input to the spinal cord, which changes during voluntary movement as compared to rest (Seki et al., 2003). Visual inputs change too. If the subject is controlling a prosthetic arm, its visual representation obviously will differ from that of a native arm, as will incorporation of the prosthesis into the internal body schema (i.e., embodiment). Even if the subject is controlling a cursor on a computer screen, the visual motion of the cursor viewed by the subject will differ, being smoother and more accurate during hand-control but showing more jitter during brain-control. In addition, many changes in cortical activity may represent adaptation on the part of the subject to fit the linear model of the BMI decoder (Wolpaw, 2010; Chase and Schwartz, 2011). More detailed scientific understanding of such changes in cortical activity that occur upon switching to brain-control may improve BMI performance.

### Transformations in the spinal cord and phases of motor control

Though classically viewed as a simple communication channel between the brain and the motor periphery, the spinal cord now is known to contain complex circuits that make important contributions to natural movements. Beyond the basic reflex pathways that can elicit movement from sensory input through only one or two synapses without cortical interaction, central pattern generators in the spinal cord can produce complex rhythmic behaviors without patterned input from the brain or feedback from the periphery (Shik and Orlovsky, 1976; Stein, 1978; Grillner, 1985). Descending signals from the brain are likely to engage parts of this spinal circuitry for production of other, non-rhythmic movements as well (Georgopoulos and Grillner, 1989). Indeed, in most mammalian species, the descending fibers of the corticospinal tract end on spinal interneurons, not motoneurons. And in macaques, which do have direct cortico-motoneuronal (CM) projections, spinal interneurons output different information (Maier et al., 1998; Fetz et al., 2002). For example, whereas most CM-cells were active selectively when subjects exerted either flexion or extension wrist forces, a high percentage of spinal interneurons were active for both flexion and extension force production, as well as at rest. With such complexity in the spinal cord, it becomes apparent that spinal cord circuitry may be doing much more than simply relaying the current input of descending cortical signals to generate muscle activity.

An emerging distinction between motor signals in the cortex vs. those in the spinal cord has to do with relative degrees of dynamic vs. static function in controlling non-cyclical limb movements. Neurons in the motor cortex typically are relatively quiescent during maintenance of a steady posture, become intensely active leading limb movement, and then show declining activity as a new steady posture is established. In large part such observations are attributable to a stronger relationship to movement velocity than to position (Moran and Schwartz, 1999). Moreover, strong rotational dynamics of joint M1 neuron firing rate trajectories may reflect a complex, dynamical system responsible for the encoding of movement (Churchland et al., 2012; Hall et al., 2014). In comparison to cortical neurons, spinal interneurons show more static activity. In monkeys generating wrist forces isometrically and auxotonically, for example, cortical neurons produced relatively transient signals for ensuing motor actions while spinal neurons generated more sustained activity, suggesting that to some degree cortical signals to change state are integrated by spinal circuitry (Shalit et al., 2012). Indeed, recent models of spinal-like regulators have demonstrated that oversimplified step inputs from the brain could be transformed by spinal circuitry to replicate much of observed center-out reaching behavior (Tsianos et al., 2014). Implementing such spinal-like circuitry in BMIs may substantially improve the quality of the transitions between movement and posture.

The transition from movement to posture is but one example of what more generally might be considered different sequential phases of motor control. A single decoder cannot be expected to deal with all phases efficiently. Cortical neurons can be identified, for example, that are active specifically in relation to rest/posture in contrast to movement (Humphrey and Reed, 1983; Williams et al., 2013; Velliste et al., 2014). Including such neural activity in the same linear decoding algorithm that drives movement velocity would be counterproductive. But if one decoder used movement-related activity to drive motion during movement phases, and another decoder used posture-related activity to maintain position during postural phases, smoother and more efficient performance might be obtained. Such control would require yet another decoding algorithm to identify transitions between phases, sometimes referred to as changes of state (Kemere et al., 2008; Aggarwal et al., 2013; Kang et al., 2015). Sequential phases or states may include not just alternation between movement and posture, but also inattention, watchful waiting for an instructional cue, preparation of a specific motor plan, reaction time following a go cue, then reaching, grasping, manipulation, and others as well. Detecting these various phases and decoding them differently may be an important step toward achieving natural human performance with BMIs (see also section More Neurons than Controlled DOFs creates a Null Space, below).

## Controlling multiple degrees of freedom

### Independent degrees of freedom

Analysis of voluntary movement consistently has shown that natural movement almost never occurs in isolation at a single joint or by activation of a single muscle, even the movement of a single finger (Hager-Ross and Schieber, 2000). For example, typists and pianists produce simultaneous movements of multiple digits even when striking a single key (Flanders and Soechting, 1992; Engel et al., 1997). Nevertheless, most of the variance of complex multi-joint movements can be reduced mathematically to a relatively small number of principal components, each of which captures a pattern of simultaneous motion at multiple joints (Santello et al., 1998; Mason et al., 2004). Such findings suggest that the number of DOFs being controlled actively during many natural movements might be less than the number of DOFs actually moving.

Bernstein (1967) first defined this problem of redundant DOFs in the musculoskeletal system: Many movements made in three-dimensional space engage more than three joint angles and more than three muscles. Mathematically, therefore, a given movement can be made in many different ways, i.e., infinite possible solutions can successfully accomplish a given movement task. Observing blacksmiths, for example, Bernstein noticed that while the joints of the arm might take quite different trajectories during a series of hammer strikes, the endpoint of contact was very consistent. This and other observations of considerable variation in certain DOFs while others are controlled precisely was formulated subsequently by Scholz and Schöner (1999) as the uncontrolled manifold hypothesis: Within the high dimensional configuration space that completely defines movements for a given task, there exists two orthogonal subspaces. Motion in a controlled subspace contains a set of actively controlled variables that are being monitored and controlled by the subject and are most important to completing the task. The other, uncontrolled subspace contains all motion orthogonal to the controlled variables and thus has no effect on successful task completion. Increased variability has been observed in the uncontrolled subspace compared to the controlled subspace in a wide variety of natural movement tasks (Scholz et al., 2001; Latash et al., 2002; Tseng et al., 2002; Kang et al., 2004), and recently in neuronal activity as well (Kaufman et al., 2014; Law et al., 2014).

Current BMI design, however, remains limited in strategies that take into account the relative importance of the various DOFs in different tasks, instead controlling the same fixed set of DOFs of the prosthetic device independently at all points in time. Likewise, regression and updating algorithms assume each DOF is encoded equally at all times. As the number of DOFs increase, BMI control becomes more difficult because more DOFs must be monitored and controlled by the subject. Models that more closely align BMI control at a given time with a subset of DOFs selected judiciously for the current task may enable more intuitive and precise control.

### Kinematic and muscular synergies

One means of selecting subsets of multiple DOFs for BMI control is to look for naturally occurring patterns of simultaneous motion at multiple joints or patterns of simultaneous activation of multiple muscles. A small number of fixed patterns of multi-joint motion (Santello et al., 1998; Mason et al., 2004) or multi-muscle activity (d'Avella and Bizzi, 2005), each varying in amplitude and timing, in theory could produce a very large repertoire of smoothly coordinated motor output. Synergies that distribute forces across the fingers can also provide a balance between flexibility and stability (Latash et al., 2007).

Indeed, synergies identified with dimensionality reduction techniques—such as principal component analysis or non-negative matrix factorization—can provide a simplified view of complex movements. Two important scientific questions are (i) whether such synergies are, in fact, used by the nervous system in controlling natural movement, and if so, (ii) in what part(s) of the nervous system the synergies are instantiated. Recent studies indicate that many fundamental synergies may be organized in the brainstem and spinal cord, rather than the cortex (Buford and Davidson, 2004; Cheung et al., 2009; Baker, 2011; Roh et al., 2011; Giszter and Hart, 2013). Once these two questions have been answered, BMI performance might be enhanced by recording from these regions and using the decoded output to drive the relevant synergies rather than the individual degrees of freedom.

Whether or not synergies are used naturally by the nervous system, performance might be improved by incorporating synergies in BMI design. One synergy already in use involves control of arm endpoint in 3 dimensions (i.e., the location of the hand) with a robotic arm that has 4 rotational DOFs: 3 at the shoulder and 1 at the elbow (Lebedev et al., 2005; Velliste et al., 2008; Hochberg et al., 2012). Rather than providing the subject with independent control of all 4 DOFs, BMI output typically drives motion of the arm's endpoint in the 3 Cartesian coordinates (e.g., horizontal, vertical, depth), and this 3-dimensional output is partitioned across the 4 rotational DOFs by a fixed subroutine. Eliminating one DOF in this manner simplifies the control task for the subject at the cost of restricting the ways in which the robotic arm can move. Further incorporation of such simplifying synergies may enable the apparent complexity of movements achieved with BMIs to grow more rapidly than the complexity of control actually required of the subject.

Some synergies might be incorporated even in device hardware. For example, in the majority of hand motion for grasping, the four fingers extend roughly in parallel to open the hand. Rather than providing separate actuators (motors and cables) to extend each finger independently, a robotic hand could have one motor with a cable that divides to attach to each finger, reducing 4 DOFs to 1. Furthermore, extension of the fingers is rarely if ever used to apply substantial forces to objects, this being accomplished with finger flexion. If the extensor cable to each finger could be elastic, then independent flexion of each finger still could be achieved by independent flexion actuators for each finger.

Synergies may be especially useful in controlling a prosthetic hand. Whereas, motion of the shoulder, elbow, and wrist to transport and orient the hand involves 7 rotational DOFs, motion of the thumb and fingers involves 22. Yet even in sophisticated uses of the hand such as typing or piano playing, rarely if ever are individual DOFs moved independently (Soechting and Flanders, 1992, 1997; Engel et al., 1997). Current BMI decoding methods nevertheless assume separate channels for each individual digit, with no relationship between them. And state-of-the-art robotic hands now provide almost as many DOFs as are found in the natural hand (Dalley et al., 2010; Johannes et al., 2011; Resnik et al., 2013; Hutchinson, 2014). For most uses of the hand in activities of daily living, current BMI systems that attempt to control all the DOFs in the hand independently may, in fact, be overly complex.

Nevertheless, identifying an optimal set of synergies for controlling a prosthetic hand is far from simple. An orthogonal basis set of the multiple joints of the hand created with dimensionality reduction has obvious advantages both in its simplicity as well as straight-forward implementation in BMI applications (Ciocarlie et al., 2007; Vinjamuri et al., 2011; Velliste et al., 2012). Alternatively, observation can be used to select a limited set of basis functions. In one recent study, a human subject grasped a variety of objects by controlling a robotic hand through four independent basis functions identified by clinical observation of hand use: (i) pinch between the thumb and index, (ii) flexion and extension of the ring and little fingers in parallel, (iii) ab/adduction of all the fingers, and (iv) opposition of the thumb (Wodlinger et al., 2015). Yet such a simple, orthogonal basis set may fail to capture certain desirable features of hand motion. For example, while the flexion/extension of all five digits is a synergy commonly identified by analysis of hand movements, the thumb and index finger also move more independently than the other three digits. Thus, difficulty arises in trying to design a simple basis set that allows for a single degree of freedom that controls the opening and closing of all digits of the hand while also allowing independent control of the thumb and index finger.

While simplifying control, fixed synergies thus necessarily limit the ability to create all of the diverse movements of which humans are capable. An alternative model by Arbib and colleagues described “virtual fingers” and a schema that added or subtracted the number of digits to a central gripping controller depending on the size and shape of the object to be grasped (Arbib et al., 1985). This virtual fingers model essentially creates different synergies depending on the context of object size and shape. Extending the use of different sets of synergies depending on the particular task or context (e.g., throwing a ball vs. typing) may advance BMI control substantially toward normal human performance. Fully independent control of all the hand's DOFs may be valuable only for the most sophisticated uses of the hand.

## More inputs than outputs

### Redundancy in neuron populations

Of the roughly 100 billion neurons in the human cerebral cortex, approximately 1.4 million send axons through the corticospinal tract to synapse on motoneurons and interneurons in the spinal cord (Lassek, 1954). In achieving the remarkable performance of normal humans, these fibers convey much of natural cortical control to the “physical plant,” which consists of approximately 600 muscles and 200 mechanical DOFs at the joints. At present, BMIs can sample only a small subset of any neuron population. As long as the sampling is reasonable, linear decoders can extract a representation of native limb motion and drive BMI end-effectors in multiple DOFs. A current trend, however, is to record increasing numbers of neural signals (Fitzsimmons et al., 2009; Lebedev and Nicolelis, 2011) with the goals not only of controlling more DOFs, but also of making the BMI more robust both to natural variability in neuron firing and to dropout of previously recorded neurons. How does the number of neurons used to control a BMI affect performance?

**Figure 2:**
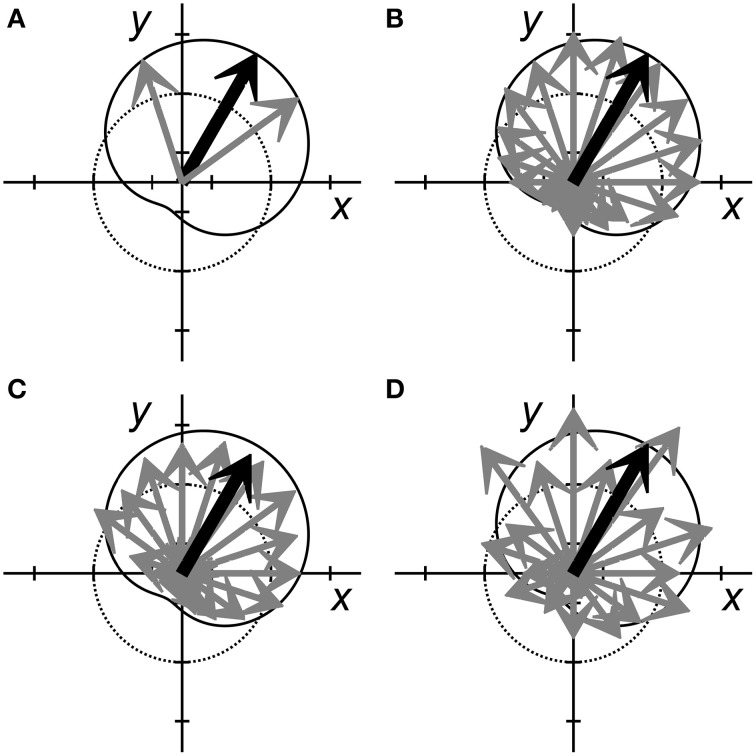
**Population vectors for neural encoding of two variables, *x* and *y***. Each neuron is represented by a gray arrow pointing in each cell's preferred direction. The length of each arrow represents the neuron's firing rate. The dotted circle represents a normalized baseline rate for each neuron, with arrow lengths greater than the dotted line representing increased firing rates and shorter lengths representing decreased firing rates. The black arrow represents the population vector sum that predicts the variables, *x* and *y*. The solid cardioid curve represents the expected firing rates of perfectly tuned neurons with various preferred directions when *x* = 0.5 and *y* = 0.87. **(A)** Two perfectly tuned neurons can encode *x* and *y* exactly, but the noise of the output will be equivalent to the noise of the two inputs. **(B)** More independently noisy neurons (here 20 neurons) can be combined to generate an accurate estimate of *x* and *y* with less noise. **(C)** When noise is correlated across neurons, adding more neurons to the population may not improve prediction. In this example, noise produced a correlated reduction in the firing rates of most neurons, resulting in a low amplitude prediction (small population vector). **(D)** In this example, although the neural activity does not match the tuning model (solid cardioid) as accurately as in **(B)**, the predicted *x* and *y* values nevertheless are similar. This departure from the tuning model for *x* and *y* might represent either increased noise, or the encoding of additional features.

In the simplest BMI, a single neuron might be used to control each DOF (Fetz and Baker, 1973; Law et al., 2014). Figure 2A schematically illustrates 2 neurons (gray arrows) controlling 2 dimensions, using the familiar population vector approach. Each gray arrow represents a different neuron, the direction of the arrow represents that neuron's preferred direction, and the length of the arrow represents its firing rate. The black arrow represents the resulting population vector, and the cardioid curve indicates the idealized linear model of cosine tuning relative to the current output, i.e., the population vector. This arrangement has a major limitation: the noisiness of the individual neurons will produce equivalent noise in the resultant output.

Increasingly precise output can be obtained by linear summation of the observed firing rates of more neurons. Figure 2B illustrates such a situation, now using 20 neurons. In an idealized linear encoding model—where all neurons have the same noise properties and are independent—adding more neurons reduces error. Regardless of the specific partitioning of linear encoding across a population of *n* neurons, the predicted error decreases as $1∕\sqrt{\text{n}}$, as follows from the central limit theorem. Adding more neurons thus progressively reduces error albeit requiring progressively increasing numbers of additional neurons for a given magnitude of reduction in error. In practice, as the number of neurons used for BMI control has increased, achieving BMI accuracy that matches idealized encoding behavior has become increasingly difficult. BMI performance tends to plateau once ~50 recorded neurons have been incorporated, after which much less improvement occurs as more neurons are added (Homer et al., 2013; Tehovnik et al., 2013).

This departure from ideal encoding results in part from the fact that neurons typically are not entirely independent of one another. Sampled neurons often have correlations related not only to the encoded signal, but also to other signals. The correlations produced by these other signals—signals that typically are unknown, unmonitored, and not experimentally controlled—constitute noise with respect to the signal being encoded (Lee et al., 1998). When the noise is correlated, even weakly as is typically observed in cortical recordings (Zohary et al., 1994), perfectly accurate linear encoding becomes impossible. Noise correlations, for example, could cause the estimate for a given trial to converge to an incorrect value. Figure 2C illustrates such an example in which a correlated decrease in firing amongst many members of a neuron population causes the magnitude of the resulting population vector to be too small. Even as more and more neurons are added to the decoded population, such noise correlations prevent converging toward perfect decoding.

As more and more neurons are added, incorporating some individual neurons to decode a given variable may actually be detrimental to BMI performance. The relative strength of various signals among different neurons may make adding some neurons corrupted by correlated noise worse than using only those with strong, independent signals. In a BMI application, the ability to generalize control from an initial regression is critical and runs the risk of overfitting if the amount of sampled data is too small for the number of neurons. Also, the ability of the user and/or the decoder to adapt and learn quickly may be diminished as the neural space that must be explored becomes larger. Recognizing such issues, algorithms have been developed to identify those neurons that provide the most independent information, permitting more parsimonious selection of neurons for input to the decoding algorithm (Singhal et al., 2010; Kahn et al., 2011; Xu et al., 2013).

Additionally, the noise levels across a population related to a given variable may not be stationary with time. Figure 2D illustrates a situation in which the individual neurons appear to be relatively noisy. Though the population vector is similar to that of Figure 2B, in Figure 2D the individual neurons appear to deviate randomly from the idealized cosine tuning model indicated by the cardioid curve. Yet this apparent noisiness might represent another signal encoded by the same neuron population (see section Selective Encoding of Variables at Different Times below). How can we make judicious choices regarding the number of neurons used for BMIs?

### More neurons than controlled DOFs creates a null space

Although the optimal trade-off between the number of neurons recorded and the number of DOFs being controlled by linear BMIs has yet to be well understood, some insight can be gained by considering the simultaneous firing rates of a population of *n* recorded neurons as an *n*-dimensional space. When used as a linear signal to control *d* DOFs, the *n*-dimensional neural signal is projected into a smaller *d*-dimensional active control sub-space, leaving a null space of dimensionality *m* = *n*–*d*. Neural activity that projects along the *m* null-space dimensions has no effect on the *d* output DOFs. If the neurons are independent predictors, then most noise tends to result in changes in this null space of the joint neural state, allowing an ensemble of “noisy” neural signals to encode the output more precisely.

Yet the null space may be more than a repository for noise. Much of what appears to be noise related to a given signal may represent neural activity related to other signals encoded by the same population of neurons. Below we explore two other potentially valuable aspects of the null space that permit: (i) motor learning with rapid flexibility, and (ii) non-linear encoding that repartitions the active control space vs. the null space depending on the phase of movement.

#### The null space and motor learning with rapid flexibility

While the null-space can be considered an “uncontrolled manifold” in a particular movement scenario (Scholz and Schöner, 1999; Latash et al., 2007), in the case of a neuronal state space, the uncontrolled manifold is not entirely uncontrolled. During both natural arm movements and BMI output, the joint neural state tends to follow a subset of preferred trajectories that use a subset of the null space, rather than using all possible trajectories distributed throughout the *n*-dimensional neural space that could provide an equivalent output (Kaufman et al., 2014; Law et al., 2014). Preferred trajectories through the neural state space may reflect the network architecture of pre-existing synaptic connections in which the neurons are embedded (Sadtler et al., 2014). Yet to control novel BMIs, monkeys can learn to use relatively novel neural trajectories (Jarosiewicz et al., 2008; Ganguly and Carmena, 2009; Ganguly et al., 2011; Law et al., 2014). A similar process of learning to use novel neural trajectories may underlie the natural process of learning new motor skills and then switching rapidly at will between one skill and another, according to the context. The relatively large number of neurons, *n* > *d*, is no longer entirely redundant when additional trajectories through the neural state space must be utilized in additional contexts. Finding and utilizing such additional trajectories might entail learning to associate a previously learned trajectory with a new context, modifying a previously learned trajectory for use in a new context, or learning an entirely new trajectory through the neural state space.

Some evidence that motor learning and rapid flexibility in various contexts involve changes in neural trajectories can be gleaned from studies that use spike-triggered averaging of EMG activity to assess functional connectivity among those neurons that provide last-order inputs to particular motoneuron pools. Broad synchrony facilitations in spike-triggered averages of EMG activity provide evidence that synchronous spikes are discharged by multiple neurons with inputs to the same motoneuron pool, indicating that groups of such neurons receive common or serial inputs (Baker and Lemon, 1998; Schieber and Rivlis, 2005). Among M1 cortico-motoneuronal (CM) cells, such spike synchronization is most common between CM-cells that have output effects in similar sets of muscles (muscle fields), suggesting that these groups of neurons may be recruited together to facilitate a particular set of muscles (Jackson et al., 2003). The prevalence of synchrony effects in M1 neurons increases with long-term training at an individuated finger movement task (Schieber, 2002), suggesting that such long-term training increases the common inputs to neurons that all input in turn to the same motoneuron pool. This change in common inputs will alter the neural trajectory during the practiced movements. Yet the size of synchrony effects also can change rapidly when novel motor behaviors are being performed (Davidson et al., 2007), suggesting that different common inputs to the population of neurons with last-order inputs to a given motoneuron pool become active in different contexts, again indicating different neural trajectories for different contexts. These rapid changes depending on context may involve processing in cortical minicolumns and corticostriatal circuits, where the level of functional connectivity (as measured by cross-correlations between simultaneously recorded spikes in layer 2/3 and layer 5 of a minicolumn, for example) has been found to vary with the type and difficulty of the task being performed (Opris et al., 2012, 2013; Santos et al., 2014). For optimal performance, BMIs will need to take into account such context-dependent changes in neuron activity and the changes in neural trajectories they represent.

#### Selective encoding of variables at different times

This idea that the firing of an individual neuron simultaneously carries representations of multiple motor parameters has been widely accepted in neurophysiological studies for some time (Humphrey et al., 1970; Thach, 1978). In general, however, neurophysiological studies make the implicit assumption that the relative weighting of the encoded motor variables remains stationary over the time course of single movements and entire sessions. The same assumption typically is made in current BMI controllers.

Yet some neurophysiological studies have indicated that the motor variables being encoded are not constant, even within a single movement. Time-resolved linear regression, for example, demonstrated that in single M1 and premotor cortex neurons, direction is represented most strongly early in the course of reaching movements, target position is represented most strongly later, and distance is represented most strongly still later in the same movements (Fu et al., 1995). Similarly, using time-resolved analysis of variance, we found recently that the firing rates of M1 neurons vary depending on location early in the course of reach-to-grasp movements, and then on the hand shape used to grasp the object later (Rouse and Schieber, 2014). Such changes in the strength of representation of different features can be viewed as rotations of the *d* controlled dimensions in the *n*-dimensional neural state space, which will repartition the *m* null-space dimensions across the time of a single movement. Hence different variables are represented more or less selectively at different phases of a single movement, and the apparent “null” space provides room for such rotations to occur.

**Figure 3:**
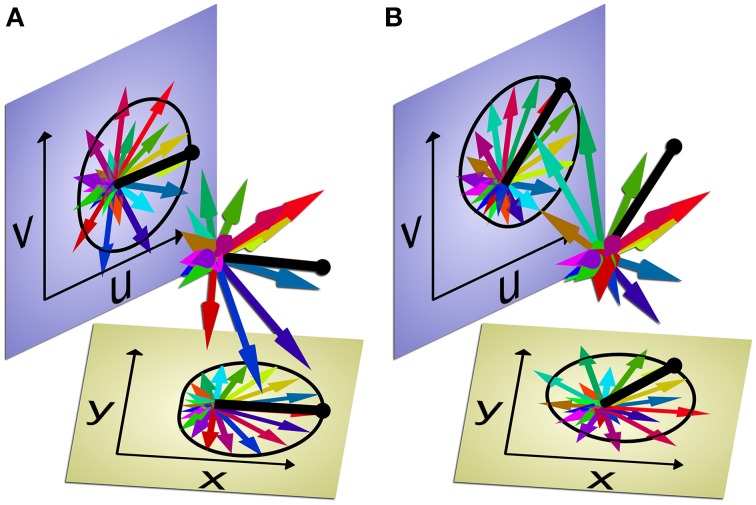
**Multi-dimensional population vector selectively encoding two pairs of variables at different times**. Each of 20 neurons is represented by a colored arrow pointing in its preferred direction. The length of each arrow represents that neuron's firing rate. The two planes, *x*⊥y (tan) and *u*⊥*v* (blue), each show the individual neuron's firing rates and preferred directions projected into the two pairs of dimensions. The population vector sum (heavy black line) projected into each plane represents the population's estimate of the two pairs of variables: *x* and *y, u* and *v*. The black cardioid curve represents the predicted firing rates given the population vector if each neuron was ideally cosine-tuned to a given pair of variables (*x* and *y*, or *u* and *v*). The two panels **(A,B)** represent two different points in time. At the first time point **(A)**, the individual neuron firing rates are related most closely to *x* and *y*, matching the idealized cosine tuning to those two variables, as represented by the cardioid in that plane. But at the second time point **(B)**, the firing rates of the same 20 neurons are related most closely to *u* and *v*. The firing rates of these neurons may not be representing both pairs of variables equally at all points in time, but rather selectively encoding one pair of variables at each time. At either time point, if a single linear decoder were used to estimate all four dimensions—*x, y, u*, and *v*—simultaneously from the population, one pair would be estimated accurately and the other pair inaccurately. But using two different decoders—one to estimate *x* and *y*, the other to estimate *u* and *v*—and then selecting the currently decoded output by assessing which idealized model is better fit at the time, would enable more accurate decoding overall. The Supplemental Video provides an animated version of this Figure. (N.B. To illustrate a 4-dimensional space in 3-dimensions, we have made *u* linearly dependent on *x* and *y* in these images; but in the actual high-dimensional neural space, all four variables can be linearly independent).

Figure 3 and the Supplemental Video both illustrate this hypothetical repartitioning of the active control space vs. the null space, again using the familiar population vector approach. As in the 2-dimensional examples of Figure 2, each arrow represents a different neuron, the direction of the arrow represents that neuron's preferred direction, and the length of the arrow represents its firing rate. In Figure 2, the *d* = 2 active dimensions were represented as the ordinate and abscissa of the plot, and the output of the single, linear model depended only on the resulting population vector sum in those two dimensions. Similarly in Figure 3A, we plot a population of 20 neurons (colored arrows) projected into the plane of two output dimensions, *x* and *y*, and the resulting population vector (black). As in Figure 2B, at the point in time illustrated in Figure 3A each individual neuron's firing rate closely matches the idealized linear model represented by the cardioid curve drawn to indicate the cosine tuning of individual neurons to the variables *x* and *y* relative to the population vector.

The remaining *m* = 20–2 = 18-dimensional null space has no direct effect on the output (population vector) of this model in this *x*⊥*y* plane. But consider the neural activity in two of these null dimensions, *u* and *v*. The neuron firing rates and resulting population vector projected in the *u*⊥*v* plane also are illustrated in Figure 3A. A different cardioid curve is shown here to represent the cosine tuning that pertains when the population vector is calculated using a second idealized model based on the projections of the neuron firing rates in the *u*⊥*v* plane. In the *u*⊥*v* plane, the firing rates of the individual neurons match this second model poorly, not unlike the example of Figure 2D.

Now consider a different point in time when the individual neurons are firing at different rates, illustrated in Figure 3B. Now the firing rates match the cardioid in the *x*⊥*y* plane poorly, but match the cardioid in the *u*⊥*v* plane well. The same neuron population that encoded *x* and *y* previously, now is encoding *u* and *v*. We hypothesize that in this manner a given neuron population may encode different variables selectively during different phases of a single movement. In such a construct, treating the 20 neuron population as a 4-dimensional output space that continuously encodes *x, y, u*, and *v* plus a 16-dimensional null space would be suboptimal. For BMI purposes, decoding different variables at different points in time would provide more accurate output for the two variables as each becomes most relevant to the current phase (or context) of movement. At each point in time, the model most heavily weighted in the BMI output could be selected by having the computer assess which idealized model is better fit by the neuron firing rates currently being generated by the brain.

Such selective neural encoding of different sets of variables at different times might be dismissed as simply reflecting an inability to create a single linear model fitting all the observed firing rates well. An inaccurate single model might result from having insufficient data (numbers of trials), insufficient numbers of neurons (e.g., *n* >> 20 is needed), or insufficient sampling of the high-dimensional parameter space (…*u, v*, …*x, y*…). Yet if a given population of neurons does indeed represent different output features at different times, applying a single linear model cannot achieve high accuracy at all points in time regardless of how many neurons or how much data are incorporated. Non-linear models will be needed to repartition the active control space vs. the null space.

As reviewed above (section The Null Space and Motor Learning with Rapid Flexibility), we envision that during natural behavior the nervous system achieves the non-linear repartitioning that selects different controlled variables by coactivating, or even synchronizing, various subpopulations of neurons at different times. In view of the wide range of inputs that impinge on a single α-motoneuron (including Ia afferents, Ia-inhibitory interneurons, excitatory spinal interneurons, and CM-cells) or that affect a single M1 neuron (including inputs from the primary somatosensory cortex, ventral premotor cortex, dorsal premotor cortex, supplementary motor area, and thalamus), we hypothesize that non-linear repartitioning may involve various inputs predominating at different times, with synaptic summation that is not necessarily linear. The extent to which the nervous system naturally uses non-linear repartitioning to output different features from the same neuron population at different times remains an open scientific question.

In any case, BMI performance might be improved by implementing methods that allow for selective encoding of the variables most relevant to the current phase of movement. This could be achieved by applying different decoding algorithms to sequentially capture different features of complex movements (Ethier et al., 2011; Jiang et al., 2011; Srinivasan and da Silva, 2011; Shanechi et al., 2012; Aggarwal et al., 2013; Kang et al., 2015). Rather than continuously decoding all DOF simultaneously, the BMI controller might use population neural activity to encode only a subset of variables while other variables are either passively dampened or even held constant by the BMI. Controlling a robotic arm and hand to reach and grasp, for example, might be improved by using neural activity sequentially, first to encode the reach location to which the arm transports the hand, and then as the hand arrives near the object, switching to encode the grasp while damping further movement of the arm. This could allow the user to focus on grasping the object precisely, without the distraction of simultaneously continuing to control the entire arm.

## Conclusions

A general theme that emerges from our considerations is that natural motor control is not a single process that applies universally in all situations. The control of small, fine movements differs from that of large, gross movements. The control of posture is not achieved by producing movement with zero velocity. Many movements may be controlled through small numbers of synergies, and only the most sophisticated performances may require individuated control of large numbers of DOFs. In generating complex movements, the same neuronal population may transmit information on different sets of output variables sequentially rather than simultaneously. To advance the performance of BMIs further toward that of normal humans will require similar strategies that go beyond one-size-fits-all, linear state space models.

Achieving such advances necessarily will require increasing the complexity of BMI controllers. Decoding will need to be more flexible and applied differently at different times, possibly driven by inputs recorded from different parts of the nervous system. Moreover, supervisory algorithms will be needed to identify the contexts and movement phases that define this dynamic relationship between neural signals and output DOFs. Implementing such designs will go further to translate our knowledge of natural motor control physiology, advancing BMIs toward normal human performance.

### Conflict of interest statement

The authors declare that the research was conducted in the absence of any commercial or financial relationships that could be construed as a potential conflict of interest.
